# Supplementary material for: In-situ study of the impact of temperature and architecture on the interfacial structure of microgels
Source: Nat Commun. 2022 Jun 29;13:3744. doi: 10.1038/s41467-022-31209-3 (PMC9243037; doi:10.1038/s41467-022-31209-3)
Supplement: Supplementary file 1 — Supplementary Information [file 41467_2022_31209_MOESM1_ESM.pdf]

Supplementary Information: In-situ study of the impact of temperature and architecture on the interfacial structure of microgels

Steffen Bochenek,[1] Fabrizio Camerin,[2,3] Emanuela Zaccarelli,[2,3] Armando Maestro,[4,5,6] Maximilian M. Schmidt,[1] Walter Richtering,[1] and Andrea Scotti*,[1]

[1]*Institute of Physical Chemistry, RWTH Aachen University, Landoltweg 2, 52056 Aachen, Germany, European Union*

[2]*CNR-ISC, Sapienza University of Rome, Piazzale Aldo Moro 2, 00185 Roma, Italy, European Union*

[3]*Department of Physics, Sapienza University of Rome, Piazzale Aldo Moro 2, 00185 Roma, Italy, European Union*

[4]*Institut Laue-Langevin ILL DS/LSS, 71 Avenue des Martyrs, 38000 Grenoble, France, European Union*

[5]*Centro de Fısica de Materiales (CSIC, UPV/EHU) - Materials Physics Center MPC, Paseo Manuel de Lardizabal 5, 20018 San Sebastián, Spain, European Union.*

[6]*IKERBASQUE—Basque Foundation for Science, Plaza Euskadi 5, 48009 Bilbao, Spain, European Union.*

* E-mail: andrea.scotti@rwth-aachen.de

Swelling Curves and Small-Angle Neutron Scattering

The microgels used in this study have been characterized in bulk using DLS and SANS. The swelling curves, R_h vs T, are reported in Supplementary Figure 1a. For comparison between the different systems we report the swelling ratio, $R_h/R_{h,40\,^{\circ}C}$, as a function of temperature (Supplementary Fig. 1b). We report the swelling curves of deuterated microgels in both H_2O and D_2O in Supplementary Fig. 1c.

From the literature,[1] a shift of the VPTT due to the deuteration of the polymer is expected and observed in Supplementary Figure 1a. Similarly, exchanging H_2O and D_2O leads to an increase of the VPTT.[2] We measured the swelling curves of the deuterated microgels in deuterated solvent. The comparison in Supplementary Figure 1c shows that at 20 °C the microgels are fully swollen and at 40 °C the microgels are collapsed. Consequently, neutron reflectivity measurements were carried out in the swollen to collapsed state of the microgels.

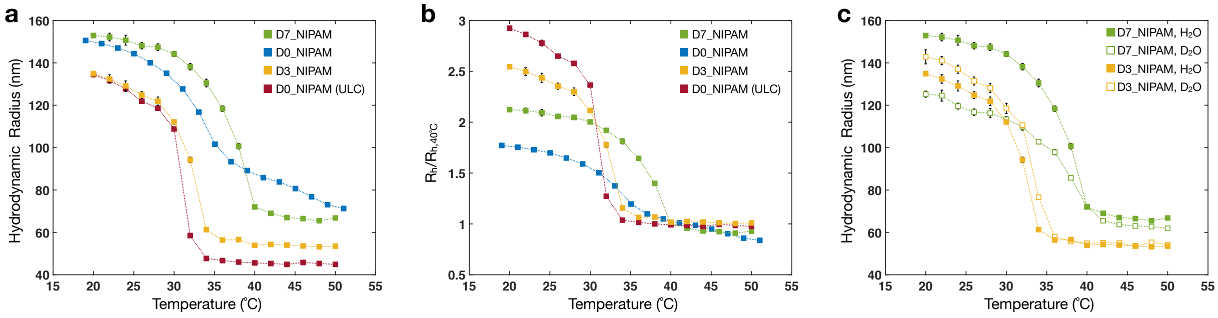

Supplementary Figure 1: Dynamic light scattering (DLS) of deuterated and hydrogenated standard and ULC microgels in water (H_2O). **a** Hydrodynamic Radius, R_h, versus temperature. **b** Hydrodynamic Radius normalized by the hydrodynamic radius at 40 °C, $R_h/R_{h,40\,^{\circ}C}$, as a function of temperature. **c** Comparison of the swelling curve of deuterated microgels (ULC D3 and 5 mol% D7) in H_2O and D_2O. The error bars are the uncertainties of the linear fits to the decay rate versus the squared scattering vector.

The internal architecture of the microgels was investigated with SANS (Supplementary Figures 2a and b). The scattering curves were fitted with the fuzzy-sphere model.[3] The results are presented in Supplementary Figures 2c and d. All microgels show the expected behaviour (see main text for more information).

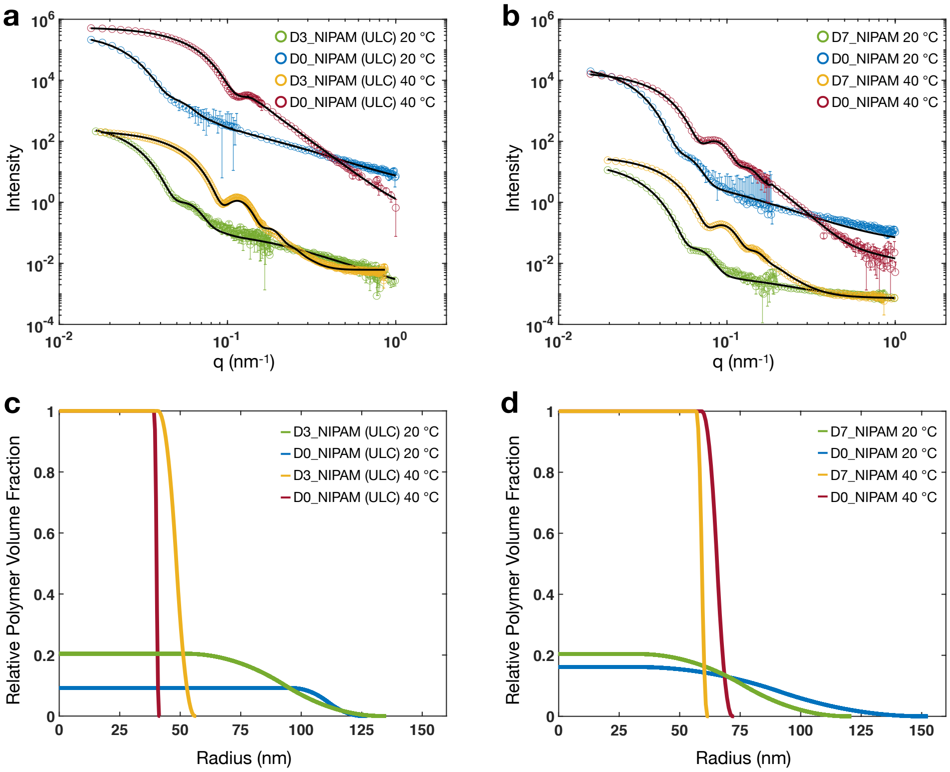

Supplementary Figure 2: Small-angle neutron scattering of deuterated and hydrogenated standard and ULC microgels in D_2O. **a** Intensity versus scattering vector, q, of ULC microgels at 20 and 40 °C with fit (black lines). **b** Intensity versus scattering vector, q, of standard microgels at 20 and 40 °C with fit (black lines). **c** Relative polymer volume fraction as a function of the radius for ULC microgels. **d** Relative polymer volume fraction as a function of the radius for standard microgels.

The characteristic lengths of the microgels are reported in Supplementary Table 1 including the errors.

Supplementary Table 1: Characteristic lengths of the individual pNIPAM based microgels below and above their VPTT with errors.

Name	T	R_h	R_{SANS}	$R_{\text{SANS,c}}$	$2\sigma_{\text{SANS}}$	$2R_{\text{2D}}$	$2R_{\text{2D,c}}$	h_{2D}
	(°C)	(nm)	(nm)	(nm)	(nm)	(nm)	(nm)	(nm)
5 mol% D0	20	(150 ± 2)	(151 ± 10)	(32 ± 2)	(119 ± 8)	(688 ± 33)	(360 ± 22)	(21 ± 2)
5 mol% D0	40	(85 ± 1)	(72 ± 4)	(59 ± 2)	(13 ± 2)	(651 ± 33)	(289 ± 15)	(26 ± 2)
5 mol% D7	20	(153 ± 2)	(120 ± 5)	(33 ± 2)	(87 ± 3)	(-)	(-)	(-)
5 mol% D7	40	(72 ± 1)	(62 ± 3)	(57 ± 1)	(5 ± 2)	(-)	(-)	(-)
ULC D3	20	(138 ± 1)	(134 ± 5)	(53 ± 2)	(81 ± 3)	(733 ± 111)	(-)	(3 ± 1)
ULC D3	40	(54 ± 1)	(56 ± 4)	(41 ± 2)	(15 ± 2)	(689 ± 134)	(-)	(4 ± 1)

Hydrodynamic radius in water, R_{h}, radius from SANS in D_2O, $R_{\text{SANS}} = R_{\text{SANS,c}} + 2\sigma_{\text{SANS}}$ where $R_{\text{SANS,c}}$ is the core radius in bulk and $2\sigma_{\text{SANS}}$ is the fuzziness of the shell in bulk determined by SANS. $2R_{\text{2D}}$ is the interfacial (dry) diameter and $2R_{\text{2D,c}}$ is the interfacial (dry) diameter of the core. h_{2D} is the maximum height once adsorbed (dry). The last three quantities are determined by AFM, see Supplementary Figs. 3 and 4. Errors are the uncertainties of the fits (R_{SANS}, $R_{\text{SANS,c}}$, $2\sigma_{\text{SANS}}$) or standard deviations ($2R_{\text{2D}}$, $2R_{\text{2D,c}}$, h_{2D}).

Single Microgels and Microgel monolayers at Interfaces

AFM phase images of deuterated ULC microgels and hydrogenated 5 mol% cross-linked microgels deposited at 20 and 40 °C are shown in Supplementary Figs. 3A, B and C, D, respectively.

These and similar images were used to determine the probability of the total interfacial diameter shown in Supplementary Fig. 4b. The corresponding AFM height images were used to obtain the averaged height profiles of the dried microgels in Supplementary Figure 4a. The averaged height profiles of the dried microgels can be compared directly to profiles extracted from the simulations, see Supplementary Figures 4c and d. In simulations, the parameter α allows to mimic the effect of temperature (see Methods). Here, for $\alpha = 0$ and 0.5, we observe that the experimentally observed behaviour of dried microgels is reproduced, see Supplementary Figure 4a. Thus, we assume that $\alpha = 0$ and 0.5 represent microgels at at 20 and 40 °C.

Combined compression isotherms with depositions can be used to investigate the properties of microgel monolayers. This procedure is explained in detail in our previous publications.[4,5] After deposition the deposited monolayers were imaged with AFM. For this, the

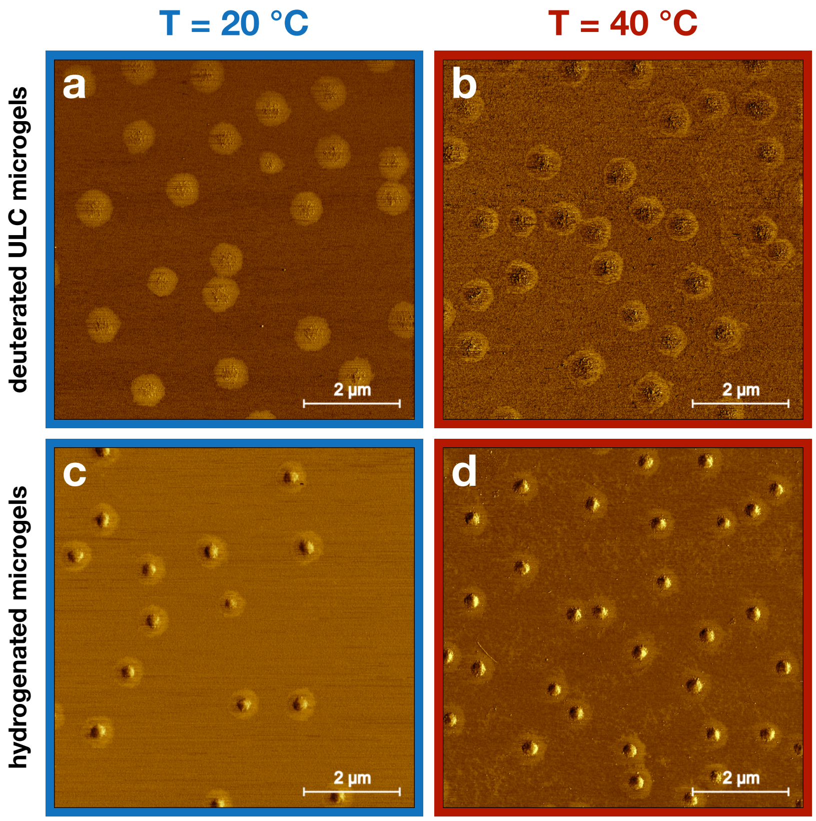

Supplementary Figure 3: AFM phase images. Deuterated ULC microgels are shown in **a** and **b** and hydrogenated 5 mol% cross-linked microgels in **c** and **d**. All microgels are deposited at 20 and 40 °C.

programmed move function of the Dimension Icon AFM was used to capture images of 7.5 x 7.5 μm (512 x 512 pixels) in a straight line along the gradient direction on the substrate every 250 or 500 μm.

The AFM images of the dried microgel monolayers were analyzed with a custom-written Matlab script based on the image analysis routine of Ref.[6] The script is wildly used in the literature to analyse images acquired with different microscopy techniques.[5,7] The script can determine the number of microgels per area, the mean nearest neighbor distances, and the hexagonal ordering parameter.

In Supplementary Figure 5 we report the influence of compression, i.e. surface pressure, on the center-to-center or nearest neighbor distance (NND) of the hydrogenated 5 mol%

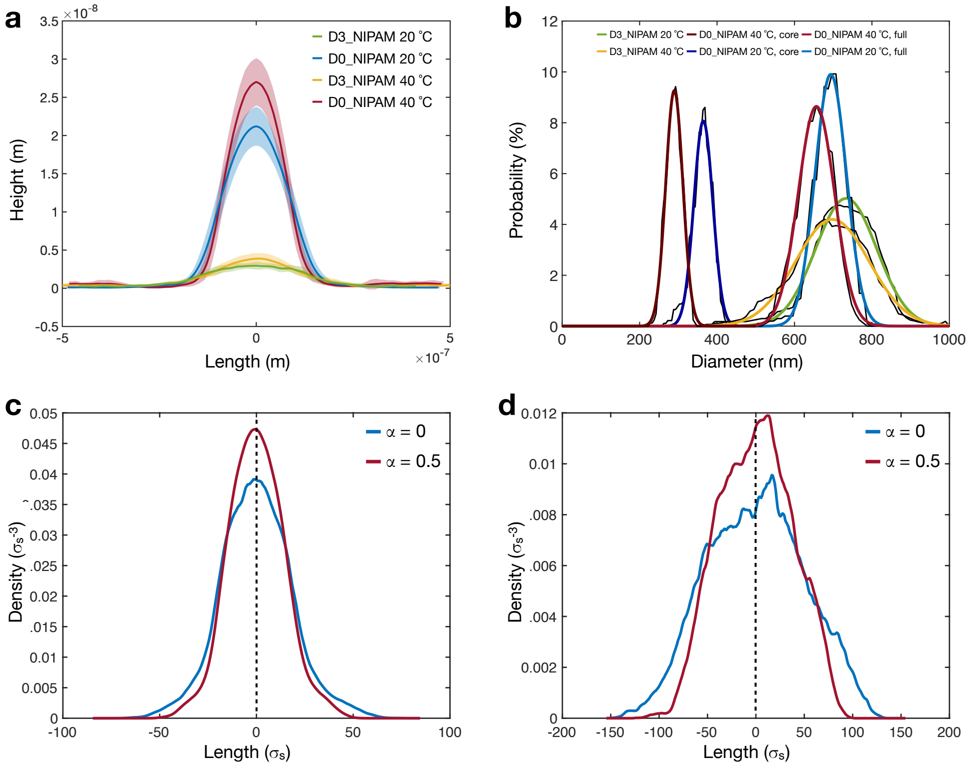

Supplementary Figure 4: **a** Mean height profiles of deposited hydrogenated 5 mol% D0 (D0-NIPAM) and deuterated ULC D3 (D3-NIPAM) microgels in dried state. Depositions were done below (20 °C) and above (40 °C) the VPTT. The error bars show the standard deviation from the image analysis. **b** Probability of the total interfacial diameter of deposited hydrogenated standard (D0-NIPAM) and deuterated ULC (D3-NIPAM) microgels in dried state below and above the VPTT. Height profiles of simulated microgels: **c** 5 mol% cross-linked microgels and **d** ULC microgels, corresponding to $\alpha = 0, 0.5, 0.9$.

cross-linked and deuterated ULC microgels. For the hydrogenated 5 mol% cross-linked microgels two temperatures, 20 and 40 °C, are reported. For the ULC microgels only 20 °C was investigated. The surface pressure region used in the neutron reflectivity experiments is highlighted by the yellow area.

Neutron Reflectivity

In Supplementary Figures 6a-c the in y-direction unshifted reflectivity curves of the three investigated NIPAM-based micrgoels are shown. At 10 and 20 °C, the curves of the 5 mol%

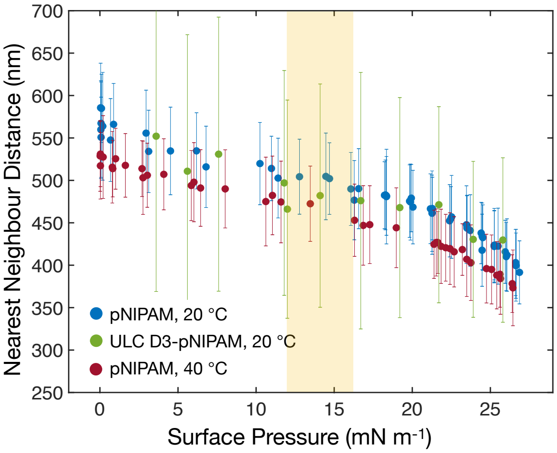

Supplementary Figure 5: Nearest neighbor distance as a function of the surface pressure for 5 mol% D0 microgels and ULC D3 microgels. For hydrogenated 5 mol% cross-linked microgels two temperatures, 20 and 40 °C are reported. The yellow area highlights the surface pressure region used in the neutron reflectivity experiments. The error bars show the standard deviation from the image analysis.

D0 microgels coincide almost entirely (Supplementary Fig. 6a). This is consistent with the swelling curves of the microgels in bulk, where well below the VPTT the size of the microgels is temperature independent.[8] However, changing the temperature across the VPTT of all three microgels leads to an increase of reflectivity at the same Q-values (Supplementary Figs. 6a-c). This increase can be attributed to an increase in density, i.e. deswelling, of the microgel monolayers.

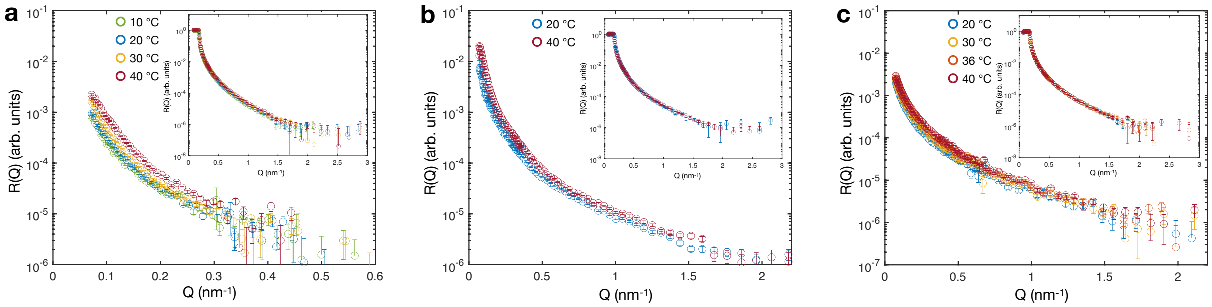

Supplementary Figure 6: Reflectivity curves of 5 mol% cross-linked and ULC microgels at different temperatures. Reflectivity curves of 5 mol% D0 microgels at the air-ACMW interface **a**. Reflectivity curves of 5 mol% D7 microgels at the air-ACMW interface **b**. Reflectivity curves of ULC D3 microgels at the air-ACMW interface **c**. Insets: Reflectivity curves at air-D_2O interfaces. The error bars represent the statistical errors on R(Q).

Supplementary Figures 7a and b depict the influence of the number of layers on the agreement of the model with the data for standard microgels. If one or two layers are used, there is no agreement with the NR data. A three-layer model shows much higher agreement, but is still far below that of a four-layer model. Comparing the SLD profiles (Supplementary Figure 7b) of the three-layer and four-layer models, we find that those of the three-layer model do not agree at all with previous experimental[9] and numerical results.[10] Therefore, the four-layer model is the best compromise between good agreement with the experimental data and additional results from other methods, and the smallest number of layers. Thus, for the standard microgels we found that $N = 4$ layers are needed to adequately describe the NR curves.

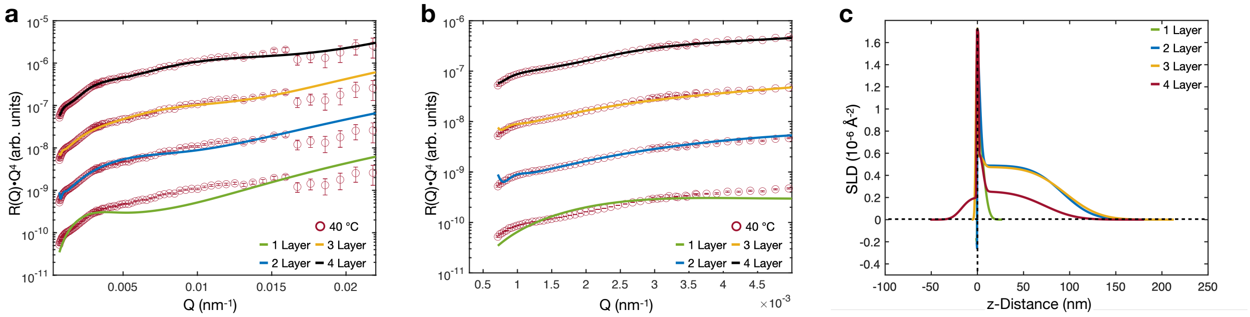

Supplementary Figure 7: **a** and **b** Reflectivity curves, R(Q)·Q^4 versus Q, of 5 mol% D7 microgels at 40 °C. The same NR data are shown four times but shifted along the y-axis. Solid lines of different colors display fits with N-layers. **b** Zoom to low Q region. **c** SLD profiles corresponding to the fits in A and B. Horizontal and vertical dashed lines are guidelines for the eyes and represent zero polymer fraction and zero z-Distance from the interface, respectively. The error bars represent the statistical errors on R(Q).

Supplementary Figures 8a-c show the SLD profiles of all microgel systems.

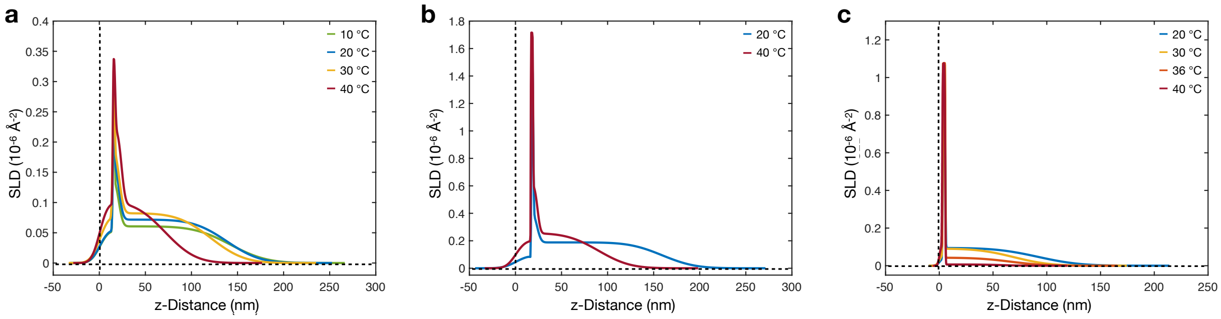

Supplementary Figure 8: SLD profiles of 5 mol% crosslinked and ULC microgels at different temperatures. **a** SLD profiles of 5 mol% D0 microgels. **b** SLD profiles of 5 mol% D7 microgels. **c** SLD profiles of ULC D3 microgels. Horizontal and vertical dashed lines are guidelines for the eyes and represent zero SLD and zero z-Distance from the interface, respectively.

Supplementary Figures 9a-c show the polymer fraction profiles of all microgel systems before they are shifted along the x-axis so that the highest polymer fraction is at a z-Distance of zero nm.

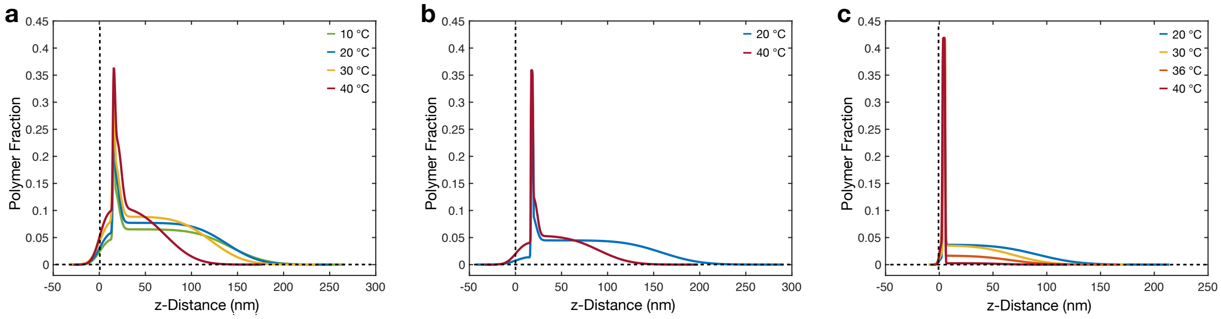

Supplementary Figure 9: Polymer faction profiles of 5 mol% crosslinked and ULC microgels at different temperatures. **a** Polymer faction profiles of 5 mol% D7 microgels. **b** Polymer faction profiles of 5 mol% D0 microgels. **c** Polymer faction profiles of ULC D3 microgels. Horizontal and vertical dashed lines are guidelines for the eyes and represent zero polymer fraction and zero z-Distance from the interface, respectively.

Parameters of model fits to the Neutron Reflectivity Data

Supplementary Table 2: Parameters of the 4-layers fit for the 5% cross-linked microgels in Figure 1 of the main part.

T	Layer 1			Layer 2			Layer 3			Layer 4			Background	
(°C)	d_1 (nm)	σ_1 (nm)	b_1 (10^{-6} Å^{-2})	d_2 (nm)	σ_2 (nm)	b_2 (10^{-6} Å^{-2})	d_3 (nm)	σ_3 (nm)	b_3 (10^{-6} Å^{-2})	d_4 (nm)	σ_4 (nm)	b_4 (10^{-6} Å^{-2})	σ_{bkg} (nm)	d_{total} (nm)
5 mol% D0 Microgels, $b_{theo} = 0.9310^{-6}$ Å^{-2}														
10	(14 ± 1)	(8 ± 1)	(0.06 ± 0.005)	(2.1 ± 0.1)	(0.7 ± 0.1)	(0.32 ± 0.06)	(4.4 ± 0.2)	(0.4 ± 0.2)	(0.14 ± 0.04)	(122 ± 3)	(3.5 ± 0.2)	(0.06 ± 0.006)	(31 ± 1)	(220 ± 6)
20	(14 ± 1)	(8 ± 1)	(0.06 ± 0.005)	(2.1 ± 0.2)	(0.7 ± 0.1)	(0.31 ± 0.08)	(4.3 ± 0.2)	(0.8 ± 0.2)	(0.19 ± 0.04)	(117 ± 2)	(3.5 ± 0.2)	(0.07 ± 0.005)	(28 ± 1)	(210 ± 5)
30	(14 ± 1)	(8 ± 1)	(0.08 ± 0.005)	(2.2 ± 0.1)	(1.0 ± 0.2)	(0.35 ± 0.06)	(4.7 ± 0.2)	(0.6 ± 0.2)	(0.20 ± 0.03)	(99 ± 2)	(4.0 ± 0.2)	(0.08 ± 0.006)	(29 ± 1)	(194 ± 4)
40	(14 ± 1)	(7 ± 1)	(0.10 ± 0.004)	(2.7 ± 0.2)	(0.5 ± 0.2)	(0.35 ± 0.05)	(6.8 ± 0.2)	(1.0 ± 0.2)	(0.23 ± 0.02)	(48 ± 3)	(3.2 ± 0.2)	(0.10 ± 0.008)	(26 ± 1)	(140 ± 5)
5 mol% D7 Microgels, b_{theo} = 4.78 10^{-6} Å^{-2}														
20	(16 ± 2)	(11 ± 1)	(0.1 ± 0.03)	(2.3 ± 0.2)	(0.5 ± 0.2)	(1.58 ± 0.01)	(3.0 ± 0.2)	(0.2 ± 0.1)	(0.49 ± 0.03)	(136 ± 4)	(3.4 ± 0.2)	(0.21 ± 0.06)	(33 ± 1)	(245 ± 14)
40	(16 ± 1)	(8 ± 1)	(0.2 ± 0.002)	(2.6 ± 0.1)	(0.2 ± 0.1)	(1.73 ± 0.005)	(4.7 ± 0.2)	(0.3 ± 0.2)	(0.62 ± 0.02)	(66 ± 1)	(2.6 ± 0.2)	(0.26 ± 0.02)	(27 ± 1)	(160 ± 2)

d_{i} is the thickness of a layer with the scattering length density b_i. σ_i is the roughness between a layer and the layer above it. d_{total} is the total film thickness and σ_{bkg} is the roughness between the last layer and the background. Errors are the uncertainties from the fits.

Supplementary Table 3: Summary of the model fits of the reflectivity curves of the ULC D3 microgels in Figure 4 in the main part.

T	Layer 1			Layer 2			Layer 3			Background	
(°C)	d_1 (nm)	σ_1 (nm)	b_1 (10^{-6} Å^{-2})	d_2 (nm)	σ_2 (nm)	b_2 (10^{-6} Å^{-2})	d_3 (nm)	σ_3 (nm)	b_3 (10^{-6} Å^{-2})	σ_{bkg} (nm)	d_{total} (nm)
ULC D3 Microgels, $b_{\mathrm{theo}} = 2.57 \cdot 10^{-6}$ Å^{-2}											
20	(3 ± 1)	(2 ± 1)	(0.04 ± 0.004)	(2.2 ± 0.2)	(0.4 ± 0.1)	(1.01 ± 0.04)	(86 ± 4)	(0.4 ± 0.2)	(0.09 ± 0.003)	(30 ± 2)	(157 ± 10)
30	(3 ± 1)	(2 ± 1)	(0.070 ± 0.008)	(2.4 ± 0.2)	(0.4 ± 0.1)	(1.08 ± 0.01)	(64 ± 2)	(0.2 ± 0.2)	(0.09 ± 0.005)	(26 ± 1)	(125 ± 5)
36	(3 ± 1)	(2 ± 1)	(0.110 ± 0.006)	(2.6 ± 0.1)	(0.2 ± 0.2)	(1.08 ± 0.08)	(61 ± 2)	(0.2 ± 0.2)	(0.05 ± 0.01)	(25 ± 1)	(120 ± 5)
40	(3 ± 1)	(1 ± 1)	(0.120 ± 0.006)	(2.7 ± 0.2)	(0.4 ± 0.1)	(1.08 ± 0.01)	(52 ± 1)	(0.4 ± 0.2)	(0.008 ± 0.004)	(15 ± 1)	(89 ± 3)

d_{i} is the thickness of a layer with the scattering length density b_i. σ_i denotes the roughness between a layer and the layer above it. d_{total} the approximated total film thickness and σ_{bkg} the roughness between the last layer and the background. Errors are the uncertainties from the fits.

Alternative fitting model of the neutron reflectivity to confirm the validity of the results obtained

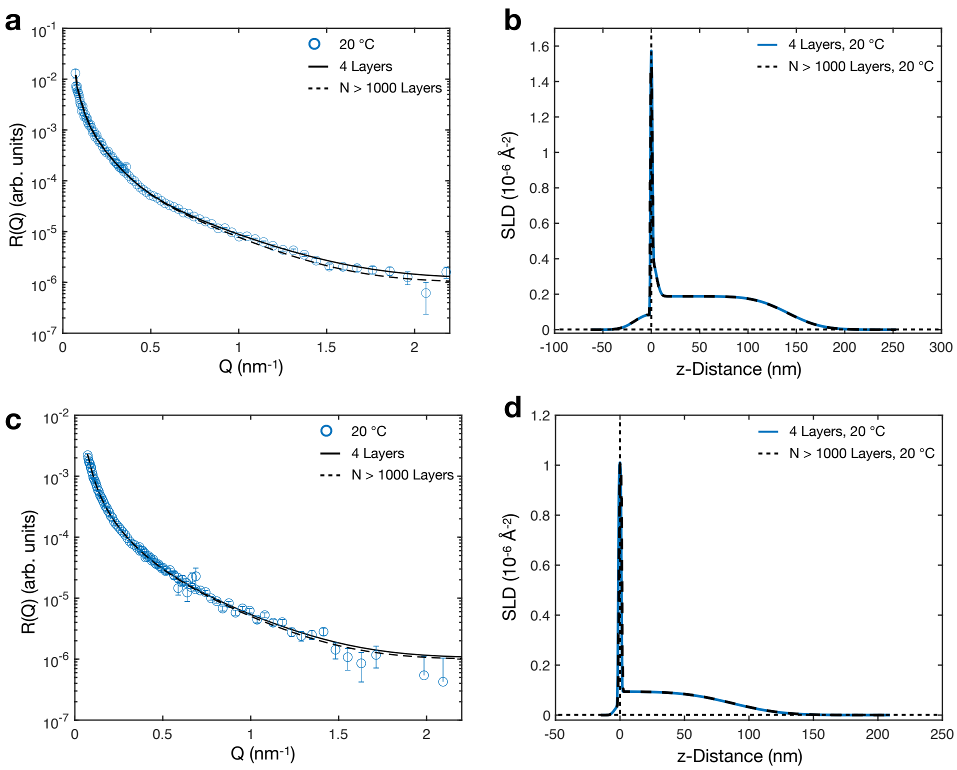

Supplementary Figure 10: Comparison between fits and the resulting SLD distributions for the deuterated 5 mol% crosslinked nanogels **a** and **b** and for the deuterated ultra-low crosslinked nanogels **c** and **d**. The data shown are relative to the swollen nanogels at 20 °C. solid lines represents the fits and the obtained SLD distribution using our multi slab model while the dashed lines corresponds to the fits with the N-layers model. The error bars represent the statistical errors on R(Q).

The interfacial structure along the z-direction of the different pNIPAM microgel studied here has been described using a model composed of N layers of varying scattering length density, SLD, (b_i) modulated by a roughness parameter (σ_i), which describes the interfacial mixing of the layers, as follows

$$b(z) = \sum_{i=0}^{N} \frac{b_i - b_{i-1}}{2} \left(1 + \mathrm{erf}\left(\frac{z - d_i}{\sigma_i \sqrt{2}}\right)\right) . \quad (1)$$

The resulting quasi-continuous model profile in all cases yielded large interfacial roughness values although smaller of the layer thickness ($\sigma_i < d_i$). Therefore, to verify that the theoretical reflectivity profiles calculated using Fresnel equations to fit the experimental data yield to physically reasonable polymer fraction profiles, two sets of data (5 mol% D7 and D3-NIPAM) were fitted using a different approach.

In detail, we confirm with an alternative fitting method that the Fresnel reflectivity calculation with the added Gaussian error function is valid and equivalent to the standard Fresnel calculation of stratified media, even when the roughness values are in the order of the layer thickness. To do so, an analytical form of the $b(z)$ profile, generated from the corresponding polymer fraction profiles, was sliced into many (N > 1000) discrete, thin layers with a thickness $t_i = 1.5$Å, a constant value of b_i and no roughness between adjacent layers (σ_i =0). Then, the SLD profile $b(z) = \sum_{i=0}^{N}(b_i - b_{i-1})/2$ was used to calculate the theoretical reflectivity by using the Parrat recursive algorithm in Motofit.

As it is shown in Supplementary Figure 10, the fits of the experimental reflectivity data using Fresnel reflectivities based on a 1000-layers model lead to the same fit quality and to the same SLD distribution as generated by our multi-slabs mode.

References

(1) Nojd, S.; Holmqvist, P.; Boon, N.; Obiols-Rabasa, M.; Mohanty, P. S.; Schweins, R.; Schurtenberger, P. Deswelling behaviour of ionic microgel particles from low to ultra-high densities. *Soft Matter* **2018**, *14*, 4150–4159.

(2) Crowther, H. M.; Saunders, B. R.; Mears, S. J.; Cosgrove, T.; Vincent, B.; King, S. M.; Yu, G.-E. Poly (NIPAM) microgel particle de-swelling: a light scattering and small-angle neutron scattering study. *Colloids and Surfaces A: Physicochemical and Engineering Aspects* **1999**, *152*, 327–333.

(3) Stieger, M.; Richtering, W.; Pedersen, J. S.; Lindner, P. Small-angle neutron scattering

study of structural changes in temperature sensitive microgel colloids. *The Journal of Chemical Physics* **2004**, *120*, 6197–6206.

(4) Bochenek, S.; Scotti, A.; Ogieglo, W.; Fernández-Rodríguez, M. A.; Schulte, M. F.; Gumerov, R. A.; Bushuev, N. V.; Potemkin, I. I.; Wessling, M.; Isa, L.; Richtering, W. Effect of the 3D Swelling of Microgels on Their 2D Phase Behavior at the Liquid–Liquid Interface. *Langmuir* **2019**, *35*, 16780–16792.

(5) Bochenek, S.; Scotti, A.; Richtering, W. Temperature-sensitive soft microgels at interfaces: air–water versus oil–water. *Soft Matter* **2021**, *17*, 976–988.

(6) Rey, M.; Fernández-Rodríguez, M. Á.; Steinacher, M.; Scheidegger, L.; Geisel, K.; Richtering, W.; Squires, T. M.; Isa, L. Isostructural solid–solid phase transition in monolayers of soft core–shell particles at fluid interfaces: structure and mechanics. *Soft Matter* **2016**, *12*, 3545–3557.

(7) Rey, M.; Hou, X.; Tang, J. S. J.; Vogel, N. Interfacial arrangement and phase transitions of PNiPAm microgels with different crosslinking densities. *Soft Matter* **2017**, *13*, 8717–8727.

(8) Brugnoni, M.; Nickel, A. C.; Kröger, L. C.; Scotti, A.; Pich, A.; Leonhard, K.; Richtering, W. Synthesis and structure of deuterated ultra-low cross-linked poly (N-isopropylacrylamide) microgels. *Polymer Chemistry* **2019**, *10*, 2397–2405.

(9) Geisel, K.; Henzler, K.; Guttmann, P.; Richtering, W. New insight into microgel-stabilized emulsions using transmission x-ray microscopy: nonuniform deformation and arrangement of microgels at liquid interfaces. *Langmuir* **2014**, *31*, 83–89.

(10) Camerin, F.; Fernández-Rodríguez, M. Á.; Rovigatti, L.; Antonopoulou, M.-N.; Gnan, N.; Ninarello, A.; Isa, L.; Zaccarelli, E. Microgels Adsorbed at Liquid-Liquid Interfaces: A Joint Numerical and Experimental Study. *ACS Nano* **2019**, *13*, 4548–4559.
